# Conditional Up-Regulation of SERCA2a Exacerbates RyR2-Dependent Ventricular and Atrial Arrhythmias

**DOI:** 10.3390/ijms21072535

**Published:** 2020-04-05

**Authors:** Bin Liu, Qing Lou, Heather Smith, Florencia Velez-Cortes, Wolfgang H. Dillmann, Björn C. Knollmann, Antonis A. Armoundas, Sándor Györke

**Affiliations:** 1 Davis Heart and Lung Research Institute and Department of Physiology and Cell Biology, The Ohio State University, Columbus, OH 43210, USA; 2 Department of Biological Sciences, Mississippi State University, Starkville, MS 39762, USA; 3 Department of Systems Biology, Columbia University Medical Center, New York, NY 10032, USA; 4 Department of Medicine, University of California, San Diego, CA 92093, USA; 5 Division of Clinical Pharmacology, Vanderbilt University School of Medicine, Vanderbilt, TN 37232, USA; 6 Massachusetts General Hospital, Cardiovascular Research Center, Charlestown, MA 02129, USA

**Keywords:** arrhythmia 1, calcium channel 2, calcium signaling 3, and ec-coupling 4

## Abstract

Ryanodine receptor 2 (RyR2) and SERCA2a are two major players in myocyte calcium (Ca) cycling that are modulated physiologically, affected by disease and thus considered to be potential targets for cardiac disease therapy. However, how RyR2 and SERCA2a influence each others’ activities, as well as the primary and secondary consequences of their combined manipulations remain controversial. In this study, we examined the effect of acute upregulation of SERCA2a on arrhythmogenesis by conditionally overexpressing SERCA2a in a mouse model featuring hyperactive RyR2s due to ablation of calsequestrin 2 (CASQ2). CASQ2 knock-out (KO) mice were crossbred with doxycycline (DOX)-inducible SERCA2a transgenic mice to generate KO-TG mice. In-vivo ECG studies have shown that induction of SERCA2a (DOX+) overexpression markedly exacerbated both ventricular and atrial arrhythmias in vivo, compared with uninduced KO-TG mice (DOX-). Consistent with that, confocal microscopy in both atrial and ventricular myocytes demonstrated that conditional upregulation of SERCA2a enhanced the rate of occurrence of diastolic Ca release events. Additionally, deep RNA sequencing identified 17 downregulated genes and 5 upregulated genes in DOX+ mice, among which Ppp1r13l, Clcn1, and Agt have previously been linked to arrhythmias. Our results suggest that conditional upregulation of SERCA2a exacerbates hyperactive RyR2-mediated arrhythmias by further elevating diastolic Ca release.

## 1. Introduction

In cardiac muscle, the release of Calcium (Ca) from the sarcoplasmic reticulum (SR) through ryanodine receptor (RyR2) channels and its subsequent reuptake by the SR Ca pump (SERCA2a) produces a transitory activation of myofibrils that underlies the heartbeat [1,2]. Dysregulated SR Ca release due to genetic or acquired defects in RyR2 has been implicated in the pathophysiology of various forms of arrhythmia and heart failure (HF) [3,4,5,6,7,8,9]. As shown for the familial arrhythmia disorder, catecholaminergic polymorphic ventricular tachycardia (CPVT) [10,11], genetic mutations in RyR2 or its auxiliary protein CASQ2, compromise the ability of the RyR2 channels to stay appropriately closed during the diastolic period, thereby resulting in aberrant, spontaneous SR Ca release. Aberrant SR Ca release results in delayed afterdepolarizations (DADs) and triggered activity, which can precipitate both atrial and ventricular arrhythmias [5,6,12]. At the same time, excessive RyR2 activity, contributes to HF by depleting the SR of Ca required for contractile activation [3,13,14]. The reasons why RyR2 hyperactivity results in these apparently different disease traits and the role of other factors, including SERCA2a Ca transport activity, in shaping phenotypic responses to enhanced RyR2 activity remain to be elucidated. 

Previous studies of the consequences of genetic upregulation of SERCA in the setting of dysregulated RyR2s have yielded conflicting results showing either detrimental [15,16] or salutary [17,18] effects on myocyte Ca handling, arrhythmia burden, and contractile function. The interpretation of these results is complicated by adaptive or maladaptive changes caused by constitutive genetic upregulation of SERCA obscuring primary consequences of combining RyR2 hyperactivity with enhanced SERCA2 function. Defining both direct and secondary consequences of SERCA2a stimulation against the backdrop of enhanced RyR2 activity is not only required for understanding the pathophysiology of Ca-dependent cardiac disease states but also has implications for the development and implementation of new therapies such as upregulation of SERCA2a in HF [19,20,21,22]. Therefore, to examine the primary effects of SERCA2 upregulation in the present study, we developed a mouse model that combines ablation of CASQ2 with doxycycline-inducible cardiac-specific overexpression of SERCA2a. Lack of substantial compensatory changes in response to SERCA2a overexpression was corroborated by broad scale RNA analysis. Our experiments demonstrated that acute upregulation of SERCA2a-mediated Ca uptake in the setting of leaky RyR2s enhances both atrial and ventricular arrhythmogenesis at the myocyte level and in vivo. 

## 2. Results

### 2.1. Acute Upregulation of SERCA2a in the CASQ2 KO Mice Did Not Alter Expression of RyR2 and Phospholamban (PLB)

**Figure 1:**
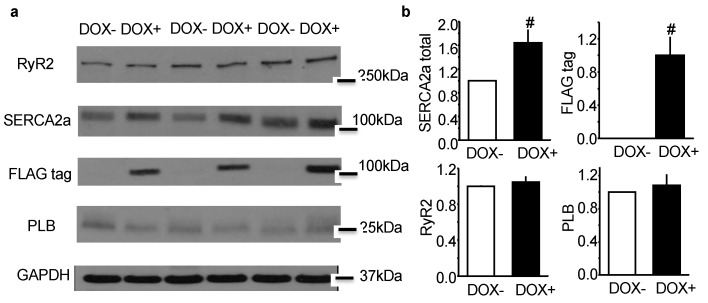
Representative western blot results (**a**) and the corresponding protein quantifications (**b**) for SERCA2a total, FLAG tag, RyR2 and PLB. Protein expression (indicated by the intensity of the band) in the DOX+ group was normalized to the paired DOX- group. 40 μg of protein lysate was loaded for each lane. Data are mean ± SEM; ^#^ *p* < 0.05 vs. DOX−.

CASQ2 KO mice were crossbred with inducible SERCA2a-TG mice to generate KO-TG mice. The induction of SERCA2a was regulated by Doxycycline (DOX), and the induced SERCA2a was distinguished from the endogenous protein by a FLAG tag. When assessed by western blotting, the overall expression of SERCA2a was upregulated by 60% in the KO-TG mice after receiving DOX (DOX+), and the FLAG-tagged SERCA2a was only detected in DOX+ mice (Figure 1). Of note, the expression of RyR2 and PLB was not altered by the induction of SERCA2a expression. Thus, our KO-TG model, while featuring DOX induced acute upregulation of SERCA2a, did not appear to show major compensatory changes; this permitted us the assessment of the direct effect of enhanced SERCA2a activity on arrhythmogenesis.

### 2.2. Acute Upregulation of SERCA2a in the CASQ2 KO Mice Exacerbated Ventricular Arrhythmias

**Figure 2:**
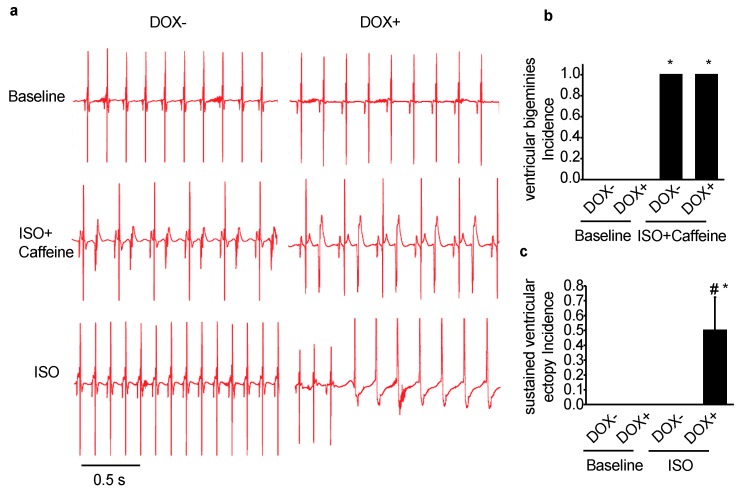
Acute SERCA2a overexpression exacerbated ventricular arrhythmias in a CPVT mouse model of CASQ2 KO. (**a**) representative ECG traces for the KO-TG mice before (DOX−) and after the induction of SERCA2a overexpression (DOX+); under baseline condition, or with injection of ISO or combination of ISO and caffeine; (**b**) the incidence of developing sustained ventricular ectopy in the presence of ISO and caffeine; (**c**) the incidence of developing ventricular bigeminy in the presence of ISO. * *p* < 0.05 vs. WT control; ^#^ *p* < 0.05 vs. DOX−.

Predisposition to ventricular arrhythmias after catecholaminergic stimulation is a hallmark of the CPVT model of CASQ2 KO mice [23]. To assess the effect of SERCA2a upregulation on ventricular arrhythmia vulnerability, ECG from the KO-TG mice was recorded before and after SERCA2a induction. Under baseline condition, overexpression of SERCA2a did not seem to affect ECG, which was normal for both DOX- and DOX+ groups (Figure 2a). In the presence of the β agonist, ISO, and caffeine, the DOX- and DOX+ mice behaved similarly, constantly displaying sustained ventricular bigeminies (Figure 2a,b), which was a marked feature of the CASQ2 KO mouse as previously described [23]. However, with the infusion of ISO alone, the DOX+ mice featured frequent bursts of sustained ventricular ectopic beats, which were not observed in the DOX- mice (Figure 2c). This data suggests that with a milder pharmacological intervention (ISO alone as compared with the combination of ISO and caffeine), the DOX+ mice exhibited exacerbated ventricular arrhythmias. Thus, acute upregulation of SERCA2a exacerbated ventricular arrhythmias in the CASQ2 KO mice.

### 2.3. Acute Upregulation of SERCA2a in the CASQ2 KO Mice Exacerbated Atrial Arrhythmias

**Figure 3:**
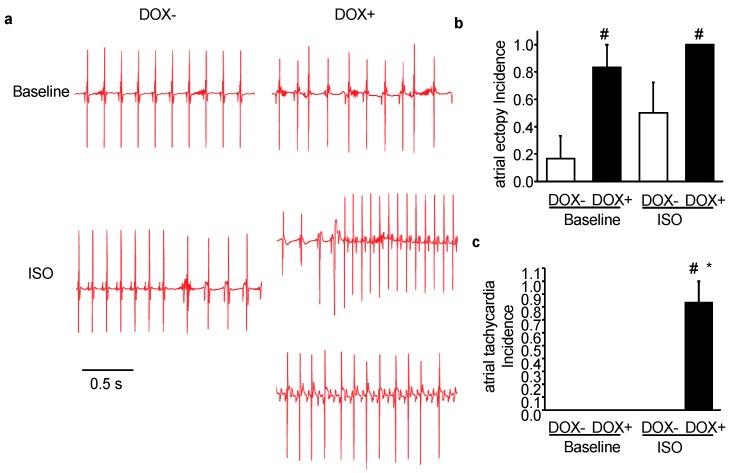
Acute SERCA2a overexpression exacerbated atrial arrhythmias in a CPVT mouse model of CASQ2 KO. (**a**) representative ECG traces for the KO-TG mice before (DOX-) and after the induction of SERCA2a overexpression (DOX+) under baseline condition or with injection of ISO; (**b**) the incidence of developing atrial ectopy; (**c**) the incidence of developing atrial tachycardia. * *p* < 0.05 vs. WT control; ^#^ *p* < 0.05 vs. DOX−.

Recent studies have demonstrated that CPVT mutations are associated with increased atrial fibrillation (AF) risk [6,24,25,26]. For instance, the CPVT model, CASQ2 KO mice were more susceptible to AF than WT after atrial burst pacing [24]. Thus, ECG from the KO-TG mice before and after receiving DOX was analyzed to assess the effect of SERCA2a upregulation on atrial arrhythmias vulnerability. Under baseline condition, the DOX- mice rarely showed atrial arrhythmias; in contrast, the DOX+ mice already displayed mild but very frequent atrial arrhythmias, featuring various atrial premature beats (Figure 3a,b). This result suggests that atrial arrhythmias were worsened even under baseline condition.

With the injection of ISO, DOX- mice displayed more atrial ectopies than DOX- at baseline, but not as much as DOX+ at baseline (Figure 3b). Compared with DOX-, the probability of developing atrial ectopy was markedly increased in DOX+ mice. Moreover, DOX+ mice also demonstrated severe atrial arrhythmias such as atrial tachycardia after ISO injection (Figure 3c). Two examples of atrial tachycardia collected from the DOX+ group were presented in Figure 3a, the regular atrial tachycardia (Figure 3a, right panel middle trace), and atrial tachycardia accompanied by conduction block (Figure 3a, right panel bottom trace). Of note, the conduction block leads to dissociation of p wave and QRS complex, but still the rapid firing of atrial ectopic foci can be determined from the high frequency of the p waves, which represents atrial depolarization. Taken together, atrial arrhythmias were also exacerbated in CASQ2 KO mice upon SERCA2a upregulation, both under baseline condition and with ISO stimulation.

### 2.4. Acute Upregulation of SERCA2a Increased Diastolic Ca Release in Intact CASQ2 KO Ventricular Myocytes

Aberrant Ca release due to hyperactive RyR2 is a hallmark feature of the CASQ2 KO myocytes [23,27]. Following β-stimulation, phosphorylated PLB leads to enhanced SERCA2a-mediated SR Ca uptake, which when combined with hyperactive RyR2, gives rise to arrhythmogenic spontaneous Ca waves (SCWs). We hypothesize that upregulation of SERCA2a in the CASQ2 KO ventricular myocytes would exacerbate aberrant diastolic Ca release by further facilitating SR Ca uptake, thus contributing to the exacerbated ventricular arrhythmias as observed in the DOX+ mice.

**Figure 4:**
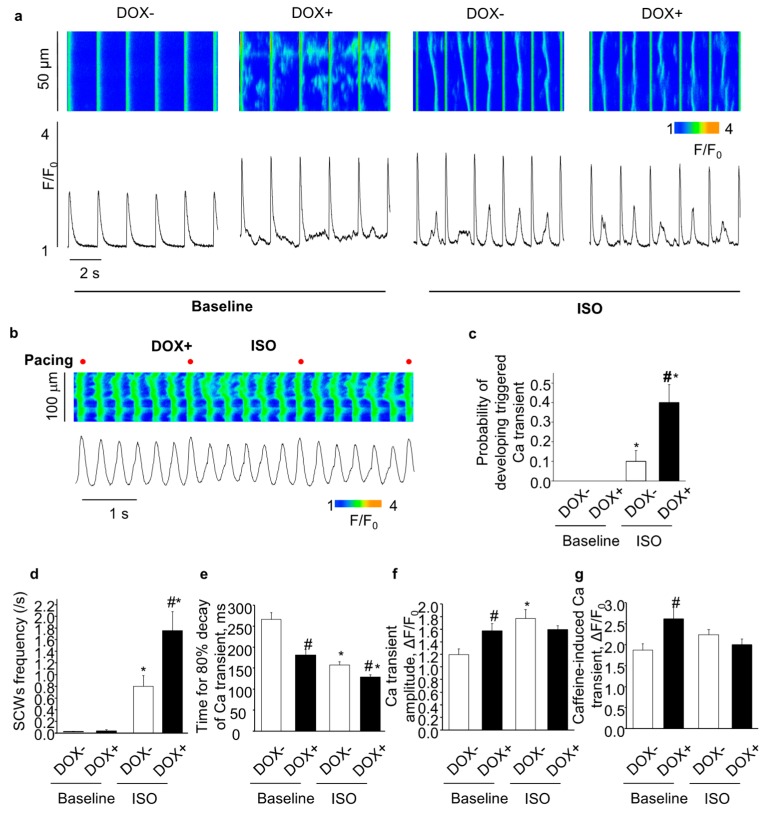
The effect of acute SERCA2a overexpression on Ca handling in intact CASQ2 KO ventricular myocytes paced at 0.5hz. (**a**) representative line-scan images (top) and time-dependent profiles (bottom) of spontaneous Ca waves (SCWs) at baseline and in the presence of 100 nM ISO; (**b**) example of triggered Ca transient in DOX+ ventricular myocytes, in the presence of ISO. Electrical stimulation is indicated by the red dot labeling; (**c**) the probability of the cells developing triggered Ca transient; (**d**) the summary of frequency of SCW; (**e**) the kinetics of 80% Ca transient decay; (**f**) amplitude of Ca transients; (**g**) amplitude of caffeine-induced Ca transients. * *p* < 0.05 vs baseline, ^#^ *p* < 0.05 vs. DOX−.

Under baseline condition, the overexpression of SERCA2a (DOX+) significantly increased the SR Ca content (Figure 4g) and the Ca transient amplitude (Figure 4f); and accelerated the Ca transient decay (Figure 4e). DOX+ myocytes did not display full scale SCWs at baseline condition. However, marking enhanced RyR2 activity, the cells showed increased frequency of sparks and wavelets (Figure 4a).

ISO infusion increased Ca transient amplitude (Figure 4f) and accelerated Ca transient decay (Figure 4e) in the DOX− cells, but did not increase SR Ca content due to the increased diastolic Ca leak (Figure 4g), which is consistent with previous studies on the CASQ2 KO myocytes [23]. Compared with the DOX−, overexpression of SERCA2a (DOX+) further accelerated Ca transient decay (Figure 4e), but did not further increase Ca transient amplitude (Figure 4f). The SR Ca content tended to decrease in DOX+ vs. DOX− (Figure 4g), which could be due to the increased diastolic Ca waves in the DOX+ cells. Indeed, SCW frequency was markedly increased in the DOX+ cells. Aside from the regular SCWs as shown in Figure 4a, DOX+ myocytes displayed enhanced frequency of cell-wide synchronized diastolic Ca release events, i.e. triggered Ca transients, evidently associated with Ca-dependent membrane excitation (Figure 4b,c). Thus, upregulation of SERCA2a increased diastolic Ca release in intact CASQ2 KO ventricular myocytes.

### 2.5. Acute Upregulation of SERCA2a Increased Diastolic Ca Release in Intact CASQ2 KO Atrial Myocytes

**Figure 5:**
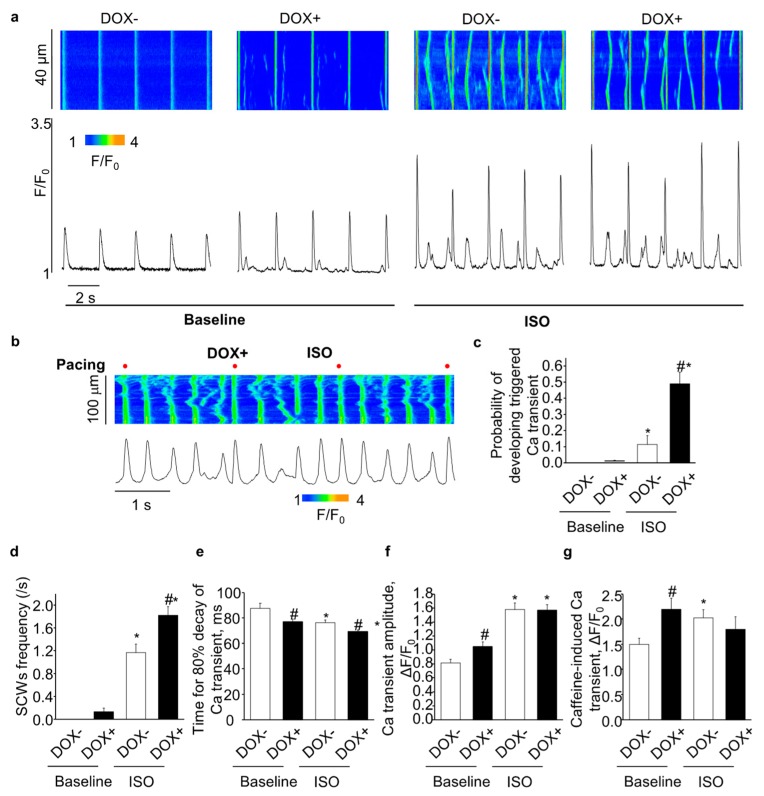
The effect of acute SERCA2a overexpression on Ca handling in intact CASQ2 KO atrial myocytes paced at 0.5Hz. (**a**) representative line-scan images (top) and time-dependent profiles (bottom) of spontaneous Ca waves (SCWs) at baseline and in the presence of 100 nM ISO; (**b**) example of triggered Ca transient in DOX+ atrial myocytes, in the presence of ISO. Electrical stimulation is indicated by the red dot labeling. (**c**) the probability of the cells developing triggered Ca transient; (**d**) the summary of frequency of SCW; (**e**) the kinetics of 80% Ca transient decay; (**f**) amplitude of Ca transients; (**g**) amplitude of caffeine-induced Ca transients. * *p* < 0.05 vs. baseline, ^#^ *p* < 0.05 vs. DOX−.

We also examined the effect of upregulation of SERCA2a on myocyte Ca handling in intact CASQ2 atrial myocytes. Under baseline condition, the overexpression of SERCA2a (DOX+) significantly accelerated the Ca transient decay (Figure 5e). In addition, similar to the ventricular myocytes, the Ca transient amplitude and SR Ca content were significantly increased in the DOX+ atrial myocytes, which also featured elevated diastolic SR Ca release manifested as Ca wavelets (Figure 5a).

ISO accelerated Ca transient decay in the DOX- atrial cells (Figure 5e), and increased Ca transient amplitude and SR Ca content (Figure 5f,g). Compared with the DOX-, overexpression of SERCA2a (DOX+) further accelerated Ca transient decay (Figure 5e), but did not change the Ca transient amplitude and SR Ca content (Figure 5f,g). Similar to the results obtained with the ventricular myocytes, SCW frequency was markedly increased in the DOX+ atrial cells (Figure 5d) due to the increased probability of developing triggered Ca transients (Figure 5b,c). Thus, upregulation of SERCA2a also increased diastolic Ca release in intact CASQ2 KO atrial myocytes.

### 2.6. Acute SERCA2a Overexpression Increased Diastolic SR Ca Release in Permeabilized CASQ2 KO Myocytes

**Figure 6:**
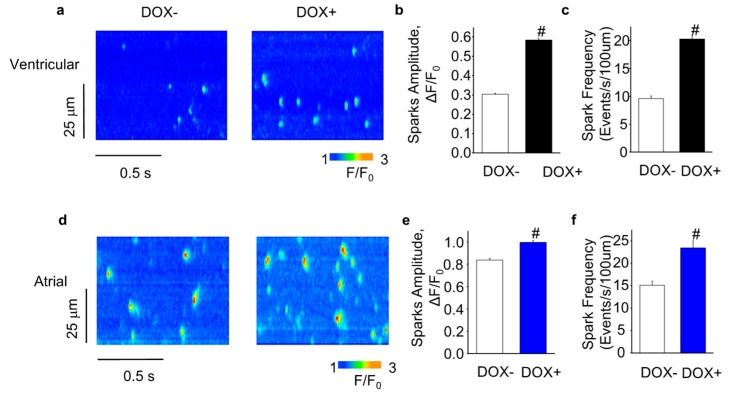
Acute SERCA2a overexpression increased SR Ca leak in CASQ2 KO myocytes. (**a**) representative line-scan images from permeabilized ventricular myocytes. Amplitude; (**b**) and frequency (**c**) of Ca sparks in ventricular myocytes; (**d**) representative line-scan images from permeabilized atrial myocytes; Amplitude (**e**) and frequency (**f**) of Ca sparks in atrial myocytes. ^#^ *p* < 0.05 vs. DOX−.

To further assess the effect of SERCA2a overexpression on SR Ca release, we measured Ca sparks in permeabilized myocytes, which reflect coordinated openings of a cluster of the Ca release channels. Consistent with our findings in intact myocytes, the permeabilized DOX+ cells (both ventricular and atrial) displayed more frequent Ca sparks than the DOX− cells (Figure 6). Moreover, the Ca sparks amplitude was also increased in the DOX+ cells (Figure 6). Thus, SERCA2a overexpression increased spark-mediated diastolic SR Ca release in permeabilized CASQ2 KO myocytes.

### 2.7. Changes in Gene Expression Induced by Acute SERCA2a Overexpression

**Figure 7:**
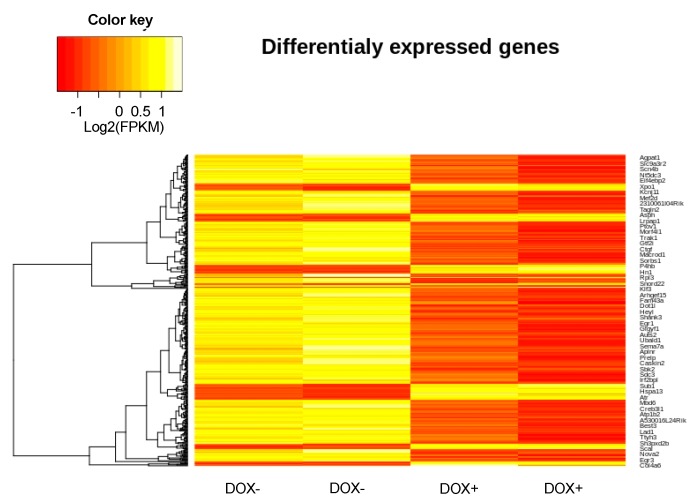
Changes in transcriptome due to acute SERCA2a overexpression in CASQ2 KO hearts. Heat map showing gene expression in hearts from KO-TG before and after induction of SERCA overexpression.

Next-generation sequencing of RNA samples extracted from mice hearts before and after induction of SERCA2a overexpression was performed to examine if altered gene expression could contribute to the exacerbated arrhythmias phenotype (Figure 7). As shown in Table 1, we detected that 17 genes were down regulated while 5 genes were up regulated upon acute SERCA2a overexpression. This relatively mild change in transcriptome was consistent with the phenotype of the KO-TG mice which did not exhibit premature death or massive remodeling that was observed when SERCA was chronically overexpressed in CASQ2 KO mice [15]. Of note, we detected significant down regulation of Ppp1r13l and Clcn1, and upregulation of Agt; the proteins encoded by these 3 genes have previously been reported to be involved in cardiac arrhythmogenesis [28,29,30,31]. Thus, changes in the expression of these genes could potentially contribute to the exacerbated arrhythmias observed in our model.

## 3. Discussion

In this study, we examined the effect of acute upregulation of SERCA2a on myocyte Ca cycling and arrhythmia susceptibility in the setting of hyperactive RyR2s using CASQ2 KO mice with doxycycline inducible overexpression of cardiac SERCA2a (KO-TG/DOX+). We found that induction of SERCA2a overexpression in CASQ2 KO mice exacerbated susceptibility to both ventricular and atrial arrhythmias characteristic of this model. Moreover, the rate of occurrence of arrhythmogenic cytosolic Ca waves increased in ventricular and atrial KO-TG/DOX+ myocytes. At the same time, SERCA2a overexpression did not result in significant compensatory changes in key cardiac Ca handling proteins (e.g., RyR2, PLB) compared to CASQ2 KO. Similarly, deep sequencing of RNA extracted from KO-TG/DOX+ vs. DOX− hearts, revealed SERCA2a induction causing only relatively minor alterations in gene expression. These results suggest that acute overexpression of SERCA2a in the setting of CPVT exacerbates arrhythmogenesis mainly due to direct effect on myocyte Ca handling.

RyR2 and SERCA2a are two principal players in myocyte Ca cycling that are modulated physiologically, affected by disease and are considered as potential targets for therapy [1,3,19,20,32,33,34]. However, how RyR2 and SERCA2a influence each other’s activities under these various conditions remains to be elucidated. In particular, previous studies have provided conflicting results about functional consequences of facilitation of SERCA- mediated SR Ca uptake in the setting of dysregulated, i.e. “leaky” cardiac RyR2s in arrhythmia and HF [15,16,17,18,35,36]. The overall deleterious effects on Ca handling and arrhythmogenesis observed in the present study with conditional overexpression of SERCA in CPVT (i.e. CASQ2-KO) mice are in agreement with those obtained utilizing constitutive SERCA1a overexpression in the same CASQ2-KO background [15]. They are also in line with the exacerbated arrhythmia and degenerative cardiac disease phenotype reported in CaMKII-overexpressing mice marked by elevated RyR2-mediated diastolic SR Ca release, upon constitutive ablation of PLB as a means to upregulate SERCA [16].

Interestingly, it has been demonstrated that combining constitutively dysregulated RyR2s (R4496C or S2814D) with ablation of PLB inhibits cytosolic Ca wave propagation and alleviates arrhythmias instead of enhancing them [17,37]. At the same time, these mice displayed increased frequency of myocyte cytosolic Ca mini-waves [17] and sparks [17,18]. Since potential compensatory changes were not addressed in these studies, it is unclear to what extent the observed alleviation of arrhythmia reflects primary or secondary responses to constitutive PLB ablation. 

In general, enhanced SR Ca uptake is expected to produce two opposing effects on Ca-induced Ca release (CICR)-mediated spontaneous Ca release known to arise and spread via a fire-diffuse-fire mechanism [38,39]: (i) stimulatory, via potentiation of RyR2 activity by luminal Ca and increased Ca flux via RyR2 secondary to increased Ca gradient across the SR membrane; and (ii) inhibitory, through rapid removal of Ca from the cytosol to the inside of the SR. Thus the differences between the two phenotypic responses (i.e. pro- vs. anti-arrhythmic) to enhanced SR Ca uptake can be attributed to a different balance between these influences resulting from either direct or secondary consequences of SERCA2a upregulation. In particular, alleviation of the arrhythmogenic phenotype could be ascribed to less pronounced sensitization of RyR2 in the heterozygous RyR2 mutation-linked model of CPVT expected to impede CICR propagation between adjacent release sites. This in turn could be either due to inherent differences in the effects of CASQ2 (or CaMKII-hyperphosphorylation) and RyR2 mutations on RyR2 channel functional activity, or secondary changes in other proteins such as Triadin, Junctin, and Histidine-rich Ca binding protein with demonstrated regulatory influences on RyR2 [27,40]. Additionally, altered (i.e. sparser) distribution of Ca release sites as a result of myocyte structural remodeling could contribute to reduced Ca wave propagation in experiments with constitutive SERCA upregulation.

Notably exacerbation of the arrhythmogenic phenotype upon SERCA2a overexpression induction developed first and was more pronounced in KO-TG/DOX+ atria, which displayed frequent atrial ectopy even at baseline only rarely seen in KO-TG/DOX- mice. The increased propensity of atria to arrhythmogenesis upon upregulation of SERCA2a could be related to differences in Ca handling characteristic of atrial vs. ventricular myocytes. In contrast to ventricular myocytes, atrial myocytes rely on propagated CICR for their normal EC coupling [41] and have a more robust SR Ca uptake and higher SR Ca load than ventricular myocytes [42]. These features, particularly the initially higher SR Ca content, would be expected to make atria more vulnerable to Ca-dependent arrhythmia upon SERCA2s upregulation.

Although RNA deep sequencing only revealed relatively minor changes in expression of RNA transcripts due to SERCA2a overexpression, changes in expression of 22 genes in the KO-TG/DOX+ vs. KO-TG/DOX− mice were detected (Table 1). Interestingly, three of these genes, *Ppp1r13l, Clcn1* and *Agt* have been previously linked to cardiac arrhythmias [28,29,30,31]. *Ppp1r13l*, which we found downregulated in KO-TG/DOX+, encodes iASPP, a regulator of desmosomes. iASPP has been reported to prevent sudden cardiac death induced by arrhythmogenic right ventricular cardiomyopathy [28]. *Clcn1*, also downregulated in KO-TG/DOX+, encodes a voltage-dependent chloride channel, which is mainly expressed in skeletal muscle, but is also present in brain and heart [43]. *Agt,* which encodes angiotensinogen, was up-regulated in KO-TG/DOX+. This gene has been associated with sick-sinus syndrome and atrial fibrillation [31,44].

In this study, we used genetic mouse models to examine the role of interplay between RyR2-mediated SR Ca leak and SERCA2-dependent SR Ca uptake in cardiac arrhythmogenesis. Compared with larger animals, including human, rodents exhibit marked difference in their cardiac electrophysiological, and intracellular Ca handling properties [45,46,47]. Thus caution must be taken in translating current findings to humans. Nevertheless, our results provide new fundamental insights into cardiac arrhythmogenesis and might be relevant for the development of novel anti-arrhythmia therapies. Specifically, our results demonstrate that acute upregulation of SERCA2a in the setting of hyperactive RyR2s exacerbates dysregulated myocyte Ca handling and arrhythmogenesis in both ventricular and atrial myocardium. These alterations are likely to reflect direct impact of upregulated SERCA activity on the Ca homeostasis affected by enhanced RyR2-mediated diastolic SR Ca release.

## 4. Materials and Methods

### 4.1. Generation of Double KO-TG Mouse Models

All animal procedures were approved by the Ohio State University Institutional Animal Care and Use Committee (2010A00000117-R3, 4/2/2019). The study conformed to the Guide for the Care and Use of Laboratory Animals published by the US National Institutes of Health (NIH Publication No. 85-23, revised 2011).

CASQ2 KO mice [23] in the C57BL/6-FVB/N hybrid background were crossbred with SERCA2a TG mice (CB6F1-Black Swiss) [48] to obtain KO-TG mice. The genotypes of the crossbred mice were confirmed by polymerase chain reactions (PCR, for CASQ2, MHC-rtTA, and Tet-RE-SERCA2a) using tail DNA. 3–6 months old male mice were utilized for experiments.

### 4.2. Electrocardiographic Recordings

Surface ECG was recorded by PowerLab 4/35, ADInstruments. After the mice were lightly anesthetized by isoflurane (1−1.5%), baseline ECG was recorded for 5 min, followed by an additional 20 min after the administration of the β-agonists isoproterenol (ISO, 1.5 mg/kg) in the absence or presence of caffeine (120 mg/Kg) via intraperitoneal (IP) injection. ECG traces were analyzed using LabChart 7 Pro (AD Instruments, Sydney, Australia). The mice utilized for the ECG recordings were not used for other experiments. 6 mice from each group (DOX− and DOX+) were utilized for the ECG recordings.

### 4.3. Cardiomyocyte Isolation and Confocal Ca Imaging

Myocyte isolation: Mice were fully anesthetized using 4% isoflurane in 95% oxygen, before surgically removing the heart. Mouse ventricular myocytes were isolated as previously described [27]. Briefly, the hearts were quickly excised and perfused on a Langendorff’s apparatus at 37 °C. After ~5 min of perfusion with nominally Ca-free Tyrode solution (containing, in mM: 140 NaCl, 5.4 KCl, 0.5 MgCl_2_, 10 Hepes, and 5.6 glucose [pH 7.3]), the perfusate was switched to Tyrode solution containing Liberase Blendzymes (0.24 U, Roche, Basel, Switzerland) for the digestion of the connective tissue. After ~20 min of digestion, single ventricular myocytes were isolated from the dissected and triturated ventricles and stabilized in Tyrode solution containing BSA (20 mg/mL). To obtain atrial myocytes, after blendzyme digestion, atrial tissue was placed in BSA containing Tyrode to cut into pieces and then gently triturated until single atrial myocytes were released into the solution.

Ca imaging: All imaging experiments were performed under room temperature. 5 mice from each group (DOX− and DOX+) were utilized for the Ca imaging experiments.

To record Ca dynamics in intact cells, the myocytes were loaded with 8 μM Fluo-3 AM (Invitrogen, Carlsbad, CA, USA) for 25 min at room temperature, followed by 25 min of incubation in fresh Tyrode solution, to wash out the dye and allow the de-esterification of the dye in the cells. Fluo-3 was excited with the 488 nm line of an argon laser and emission was collected at 500–600 nm. Fluo-3 fluorescence was recorded in the line-scan mode of the confocal microscope (Fluoview 1000 (Olympus, Tokyo, Japan) and A1R confocal microscope (Nikon, Tokyo, Japan) utilized for ventricular and atrial myocyte experiments respectively), utilizing a 60×, 1.4 numerical aperture oil immersion objective. The myocytes were paced using extracellular platinum electrodes. Cells were perfused with 100 nM of ISO to examine its effect on myocyte Ca handling, recordings started after 3 min of ISO perfusion and ISO was present during the whole recording. To assess the SR Ca load, 20 mM caffeine was applied at the end of the experiments, the caffeine induced Ca transient was used as an estimate of SR Ca content.

To record Ca sparks in permeabilized cells, fluo 4 pento potassium salt (16.2 μM) (Invitrogen, Carlsbad, CA, USA) was used. The myocytes were permeabilized with saponin (0.01% for ∼30 s) dissolved in the internal solution, which contained (mM): 120 potassium aspartate, 20 KCl, 0.81 MgCl_2_, 1 KH_2_PO_4_, 0.5 EGTA, (free [Ca] 100 nm), 3 MgATP, 10 phosphocreatine, 20 Hepes (pH 7.2), and 5 U mL^−1^ creatine phosphokinase [49].

### 4.4. Western Blots

Mouse cardiac homogenates were prepared as previously described [49]. Proteins were separated by SDS–PAGE (5–15% gradient gel, Bio-rad Laboratories, Hercules, CA, USA) and transferred onto nitrocellulose membrane. Membranes were probed by primary antibodies against RyR2 (Thermo Fisher Scientific, Waltham, MA, USA), SERCA2a (Santa Cruz Biotechnology, Dallas, TX, USA), PLB (custom), and Flag tag (Cell signaling), followed by secondary anti-mouse or anti-rabbit antibodies. SuperSignal chemiluminescence (Pierce Biotechnology Inc., Rockford, IL, USA) was utilized to detect the horseradish peroxidase conjugated protein bands. ImageJ was utilized to quantify the protein expression levels. 4 mice from each group (DOX− and DOX+) were utilized for the experiment.

### 4.5. RNA Extraction, Library Construction and Sequencing

RNA was extracted from freshly harvested mouse heart tissue by Trizol Reagent, following the manufacturer’s protocol (Thermo Fisher Scientific, Waltham, MA, USA). 3 mice from each group (DOX− and DOX+) were utilized for RNA extraction.

Libraries were constructed by a fee-based core facility at Ohio State University following the established protocol [50]. All the libraries were analyzed and quantified with a bioanalyzer and sequenced on an Illumina HiSeq 2500 system. All sequence data of the 6 libraries were deposited in the NCBI Sequence Read Archive (SRA) database under the accession number PRJNA623119.

### 4.6. RNA-seq Read Processing, Transcript Assembly, and Differential Expression

Trimmomatic was utilized to process paired-end RNA-Seq reads to remove adapters, as well as low-quality bases [51]. Trimmed reads less than 40 bp were discarded. The remaining high-quality reads were subjected to rRNA sequence removal by aligning to an rRNA database [52] using Bowtie2 [53], and up to three mismatches were allowed.

The resulting read pairs were aligned to the mm10 genome, with up to two mismatches allowed, using Tophat2 [54]. Cufflinks was used to assemble only the aligned read pairs with no mismatch into transcripts [55]. The expression level of the transcripts was measured and normalized to the number of fragments per kilobase of exon per million mapped fragments (FPKM), based on all mapped read pairs, using Cuffnorm, a program included in the Cufflinks package. Differential expression analysis was performed using Cuffdiff, also included in the Cufflinks package. Protein-coding genes with adjusted *p* values of < 0.05 and no less than two-fold changes were considered differentially expressed.

### 4.7. Statistical Analysis

Results are expressed as Mean ± SEM. Statistical significance was determined using unpaired student t test. A *p* < 0.05 was considered as statistically significant.

## Figures and Tables

**Table 1:** Changes in gene expression due to acute SERCA2a overexpression in CASQ2 KO hearts.

**Down-Regulated**	**Gene**	**log2Fold Change**	**Adjusted *p* Value**
	Fos	−2.7	1.17 × 10^−28^
	Vaultrc5	−1.7	1.85 × 10^−18^
	Atf3	−2.1	2.20 × 10^−12^
	Ppp1r13l	−1.7	2.24 × 10^−12^
	Egr1	−1.5	2.75 × 10^−11^
	Grin2c	−1.9	5.97 × 10^−11^
	Nr4a3	−1.8	6.86 × 10^−11^
	H2-T23	−2	5.71 × 10^−9^
	H2-Eb1	5	5.90 × 10^−8^
	Rn45s	−2	5.47 × 10^−7^
	5730408K05Rik	−1.7	7.19 × 10^−6^
	Ifltd1	−2.6	5.70 × 10^−5^
	Clcn1	−1.5	0.004
	Prr5	−1.6	0.004
	Rprl3	−9.7	0.006
	Mir3113	−1.5	0.01
	Nr4a1	−1.9	0.028
**Up-regulated**	**Gene**	**log2Fold Change**	**Adjusted *p* Value**
	Col4a6	4.5	3.61 × 10^−29^
	Zdhhc2	1.6	1.17 × 10^−28^
	Agt	2.2	1.19 × 10^−21^
	Rps15a-ps6	1.5	0.001
	Selenbp2	2.4	0.004

## Abbreviations

AF	atrial fibrillation
CAMKII	Ca2+/calmodulin-dependent protein kinase II
Ca	calcium
CASQ2	calsequestrin 2
CICR	calcium-induced calcium release
CPVT	catecholaminergic polymorphic ventricular tachycardia
DAD	delayed afterdepolarizations
DOX	doxycycline
HF	heart failure
KO	knock out
ISO	isoproterenol
PLB	phospholamban
RyR2	ryanodine receptor 2
SR	sarcoplasmic reticulum
SERCA	SR Ca ATPase
SCW	Spontaneous calcium waves
WT	wild type
